# Zero‐fluoroscopy catheter ablation for atrial fibrillation: a transitional period experience

**DOI:** 10.1002/joa3.12448

**Published:** 2020-10-30

**Authors:** Myung‐Jin Cha, Euijae Lee, Seil Oh

**Affiliations:** ^1^ Departments of Internal Medicine Seoul National University Hospital Seoul South Korea

**Keywords:** atrial fibrillation, catheter ablation, intracardiac echocardiography, radiation, zero fluoroscopy

## Abstract

**Background:**

Radiofrequency catheter ablation for atrial fibrillation (AF) without using fluoroscopy has been getting popular. In this study, we reported the transition period experience of the zero‐fluoroscopy procedure by an experienced operator and shared our zero‐fluoroscopy protocol.

**Method:**

A total of consecutive 30 AF ablation cases attempted to be treated without fluoroscopy were investigated. Ten serial cases were grouped as fluoroscopy‐guided period, and period 1‐3 in chronological order. All zero‐fluoroscopy attempted cases were assisted with an intracardiac echocardiography device with a three‐dimensional electroanatomical system.

**Results:**

Complete zero‐fluoroscopy procedure was achieved at the 6th case during the transitional period. During the first period, the total procedure time slightly increased in, but afterward, procedure time was continuously decreased, and it became significantly shorter in the third period than the previous fluoroscopy‐guided period. Any additional use of fluoroscopy during the transitional period was mainly for transseptal puncture and diagnostic catheter placement into the coronary sinus. Pulmonary vein isolation was achieved in all patients, and there was one case of hemodynamically insignificant moderate amount pericardial effusion.

**Conclusion:**

For an experienced operator, complete zero‐fluoroscopy AF ablation might be achieved safely and feasibly within 5‐10 cases. Fluoroscopy equipment backup might be useful during the learning period for beginners in the zero‐fluoroscopy procedure.

## Graphical abstract

The complete zero‐fluoroscopy radiofrequency catheter ablation for atrial fibrillation is feasible and safe. The learning period is not too long, and a complete zero‐fluoroscopy procedure was achieved in the 6th case. Pulmonary vein isolation was achieved in all cases, and the total procedure time was shorter than the conventional fluoroscopy‐guided procedure period.

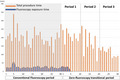

## 1 INTRODUCTION

In cardiac electrophysiology procedures for complex arrhythmias, both patients and physicians are exposed to a considerable amount of radiation.^1^ According to the “as low as reasonably achievable” principle,^1^ physicians have continuously investigated how to reduce radiation exposure in invasive cardiac procedures.^2^, ^3^ With the yearly advances in various medical tools, including the three‐dimensional (3D) electroanatomical mapping system and intracardiac echocardiography (ICE) device, successful attempts to reduce radiation exposure have been achieved.^4^, ^5^

Accordingly, the safety and efficacy of a nonfluoroscopy technique for atrial fibrillation (AF) catheter ablation have been recently reported in various studies.^6^, ^7^, ^8^ Despite the reported favorable results, there are several concerns about the zero‐fluoroscopy procedure.^9^ Near‐zero or zero‐fluoroscopy procedures are becoming increasingly popular because of the development of new technologies. In a randomized trial, ICE and the CARTO® 3 electroanatomical mapping system with contact‐force sensors, when used together, enabled the operators to achieve zero‐fluoroscopy time during left atrial mapping and ablation without prolongation of the procedure time, with similar efficacy and safety profile as conventional fluoroscopy‐guided procedures.^10^

Fluoroscopy‐guided AF procedures have existed for a long time, and in recent years, complications because of radiation exposure have been largely reduced by the use of pulsed fluoroscopy with low frame rates and maximal collimation.^11^, ^12^ On the other hand, the safety of the zero‐fluoroscopy procedure has not yet been fully demonstrated.^13^ There are difficulties in changing the existing procedural protocol because of the risks that may arise during the transitional period of the new technique, considering its risks and benefits.

In this study, we reported a single‐center experience during the transitional period from the conventional fluoroscopy to compete zero‐fluoroscopy period with the initial serial 30 cases using CARTO‐3 mapping system. Furthermore, we also aimed to describe our procedure protocol how each step was performed without fluoroscopy exposure.

## 2 METHODS

### 2.1 Patient population

A total of 30 serial cases of AF catheter ablation intended for the zero‐fluoroscopy procedure, were analyzed. Patients were divided into three groups according to the order of the procedure. The first 10 patients were grouped as “period 1," the next 10 patients as “period 2,” and the last 10 patients as “period 3” in chronologic order. Procedure outcome was compared to the most recent serial AF cases (n = 30) with conventional fluoroscopy technique before the transitional period. To minimize the interoperator bias, only one operator (MJC) with the same first assistant physician participated in the procedure during the study period.

All 30 patients had symptomatic paroxysmal or persistent AF, and documented failure of or intolerance to at least one antiarrhythmic drug. All patients continued their use of oral anticoagulants except on the day of the procedure. All antiarrhythmic drugs were discontinued >1 week before the ablation. This study was based on the experience of a single tertiary center performing around 150 AF ablation procedures annually. Procedure‐related data were collected in the same manner according to the form determined by the institution and were reviewed retrospectively for the study.

### 2.2 Preprocedural preparation

All patients underwent a preprocedural cardiac computed tomography (CT) scan within 12 hours before the procedure for evaluating abnormal intracariac structure, thrombus, or coronary artery stenosis. All patients in our series were planned to undergo an ablation procedure with the fluoroless approach. Nevertheless, a fluoroscopy system was prepared for its immediate use when needed. An electrophysiology laboratory system fully equipped with the CARTO® 3 electroanatomical mapping system (Biosense‐Webster, Diamond Bar, CA, USA), with the Confidence® module for CARTOSOUND®, CARTO VISITAG®, and CARTO VISITAG® with Ablation Index, was used.

### 2.3 Zero‐fluoroscopy procedure

#### 2.3.1 Vascular and intracardiac access

Two vascular accesses were obtained only in the right femoral vein using a modified Seldinger technique for sheath placement (one long wire and one short 8‐Fr sheath). The ICE catheter (SOUNDSTAR®, Biosense Webster) was carefully introduced into the femoral vein via the short sheath and advanced to the inferior vena cava while observing the vessel lumen. During catheter advancement, the direction could be visualized on CARTO® 3. For long‐wire advancement to the superior vena cava, the long wire can be visualized by ICE (Figure S1).

#### 2.3.2 ICE‐guided transseptal puncture

The ablation catheter was introduced into the left innominate vein guided by contact force, and the catheter direction was visualized in the mapping screen. Then, the SL‐1 sheath was advanced until a sheath error sign was detected (Figure S2). Then, the ablation catheter was pulled out and a transseptal needle (Brockenbrough needle; Medtronic) with a dilator at the 4–5 o’clock position was advanced. The ICE catheter should be fixed to show the fossa ovalis, aortic root, and superior vena cava, in order to visualize the downward movement of the sheath and the dilator (Figure S3A and Video S1). The operator can see the sheath system approaching the right atrium on the screen of the ICE device, and feel the beating heart through the fingertips of the right hand. When the sheath system is entered into the fossa ovalis, the septum is pushed and tented by the system (Figure S3B). By rotating the ICE catheter, it is possible to determine whether the puncture site is close to the anterior or posterior rim. Finally, the Brockenbrough needle was advanced to puncture the septum.

#### 2.3.3 Cardiac structure marking on the 3D image

It is useful to mark the location of the septal puncture site and left atrial wall and the esophageal geometry on the CARTO‐SOUND image (Figure S4). The left atrial volume is most accurate when measured on the ICE image because this image shows the left atrium in real‐time without any catheter pushing. If the atrium is markedly dilated, the left atrial ICE view may not be useful in demarcating the important structures. Therefore, it is useful to advance the ICE catheter into the left atrium and map the left atrial wall.

#### 2.3.4 Coronary sinus catheter placement

Through the sheath used for the ICE catheter, the diagnostic catheter was advanced to the right atrium. We usually use only one duodecapolar catheter (DuoDeca Livewire™; Abbott Laboratories) for the right atrium and the left atrium. The catheter can be visualized on the screen, and the diagnostic catheter can be easily introduced into a desirable location (Figure S5).

#### 2.3.5 Pulmonary vein isolation

Ablation was performed using a Navistar Thermocool catheter (Biosense Webster) guided by a CARTO® 3 3D navigation system. The ablation index‐guided ablation with the VISITAG system was used. The fast electroanatomical map was merged with the CARTO‐SOUND image. Based on the merged image, pulmonary vein isolation was performed during AF or sinus rhythm according to the patient’s initial rhythm. Wide area circumferential ablation was done with a default power of 35 W, with limited ranges of 25‐35 W for the posterior wall and 30‐45 W for other regions of the left atrium, to obtain an ablation index of >350 for the posterior wall and >450 for the other regions of the left atrium.

#### 2.3.6 Ripple mapping‐guided checking for complete isolation

Sinus rhythm was restored with internal electrical cardioversion when the patient’s rhythm was still AF after pulmonary vein isolation. Left atrial mapping under sinus rhythm was carefully obtained with the use of a 20‐pole high‐density mapping catheter (PENTARAY, Biosense Webster). This 5‐splined high‐density mapping catheter has four electrodes on each spline, with an interelectrode spacing of 2–6–2 mm. Mapping of all the left atrial endocardial surface and the pulmonary vein was done. The residual potential immediately after ablation was confirmed using the ripple map, and additional ablations were done on the lesion (Video S2). After 30 min from complete pulmonary vein isolation, electroanatomical mapping during sinus rhythm was performed again to check for early reconnection. The ripple map displays bipolar electrograms at each point acquired on CARTO® 3 as a dynamic bar at its 3D location, the height of which reflects the magnitude of the bipolar voltage of the electrogram at that time point in the annotation window. The unique visual representation of bipolar voltage assists in the identification of multicomponent signals and associated activation patterns. In the ripple map, the threshold of bipolar voltages for display as a dynamic bar was set at 0.03‐0.05 mV, which prevented baseline electrical noise from being displayed on the map.

### 2.4 Outcome

Any kind of procedure‐related complications was counted as safety outcomes, including procedure‐related stroke or bleeding, cardiac tamponade, or vascular complications. The feasibility was evaluated according to acute pulmonary vein isolation, residual potential, and early reconnection. A total procedure time, a total ablation time, a fluoroscopy time, and a time for transseptal puncture were recorded.

## 3 RESULTS

### 3.1 Patient characteristics

As described in Table 1, a total of 30 patients (mean age, 63 years; 57% men and 50% with paroxysmal AF) with zero‐fluoroscopy procedure were included in the analysis. The patient characteristics of 30 comparator cases (conventional fluoroscopy technique) were not significantly different from study cases. Pulmonary vein isolation was successful in all cases, and 16 (53.3%) patients underwent additional linear ablation at cavotricuspid isthmus or left atrium.

**TABLE 1:** Patient characteristics

	Zero‐fluoroscopy (N = 30)	Fluoroscopy guided (N = 30)	*P*‐value
Age (year)	62.6 ± 10.9	65.8 ± 9.8	.241
Male sex	17 (56.7%)	20 (66.7%)	.596
Height (cm)	163.7 ± 12.8	165.3 ± 11.1	.667
Body weight (kg)	66.3 ± 12.9	69.6 ± 16.7	.429
Paroxysmal AF	15 (50%)	10 (33.3%)	.295
Past medical history			
Hypertension	17 (56.7%)	12 (40.0%)	.386
Diabetes	7 (23.3%)	6 (20.0%)	.897
Myocardial infarction	3 (10.0%)	0 (0.0%)	.239
Stroke	3 (10.0%)	0 (0.0%)	.395
Heart failure	3 (10.0%)	10 (30.0%)	.153
Chronic kidney disease	2 (6.7%)	6 (20.0%)	.328
Liver disease	2 (6.7%)	0 (0.0%)	.659
Open heart surgery	1 (3.3%)	0 (0.0%)	.999
Previous PCI	3 (10.0%)	0 (0.0%)	.395
Echocardiography			
Left atrial diameter	45.1 ± 6.2	44.6 ± 7.3	.745
Left atrial volume	87.7 ± 34.7	82.9 ± 22.7	.452
Left ventricular ejection fraction	59.0 ± 6.4	57.3 ± 6.2	.364

Abbreviations: AF, atrial fibrillation; PCI, percutaneous coronary intervention; RFCA, radiofrequency catheter ablation.

### 3.2 Procedural safety and feasibility outcome

There was one moderate amount of pericardial effusion event in the 15th patient of zero‐fluoroscopy cases. However, there were no procedure‐related stroke or bleeding events (Table 2). All pulmonary vein isolations were achieved in all 30 patients. After 30 minutes of observation following pulmonary vein isolation, the number of early reconnection was 0.3 ± 0.5 in the zero‐fluoroscopy group (0.1 ± 0.3 in the fluoroscopy group). There was no significant difference between the two groups.

**TABLE 2:** Procedure outcome

	Zero‐fluoroscopy (N = 30)	Fluoroscopy guided (N = 30)	*P*‐value
Time for			
Total procedure	163.9 ± 59.7	204.1 ± 43.3	.005
Total ablation	46.8 ± 16.4	53.2 ± 13.3	.154
Transseptal puncture	7.1 ± 4.7	9.8 ± 7.2	.153
Fluoroscopy	3.7 ± 9.6	24.7 ± 10.0	<.001
Procedure outcome			
Procedure‐related complication*	1 (3.3%)^b^	1 (3.3%)^a^	.999
Complete pulmonary vein isolation	30 (100%)	30 (100%)	N/A
Additional linear ablation	16 (53.3%)	8 (26.7%)	.181
Early reconnection (per patient)	4 (14.8%)	3 (10.0%)	.999

*Procedure‐related complication included ^a^puncture site hematoma and ^b^pericardial effusion.

### 3.3 Transitional curve

**FIGURE 1:**
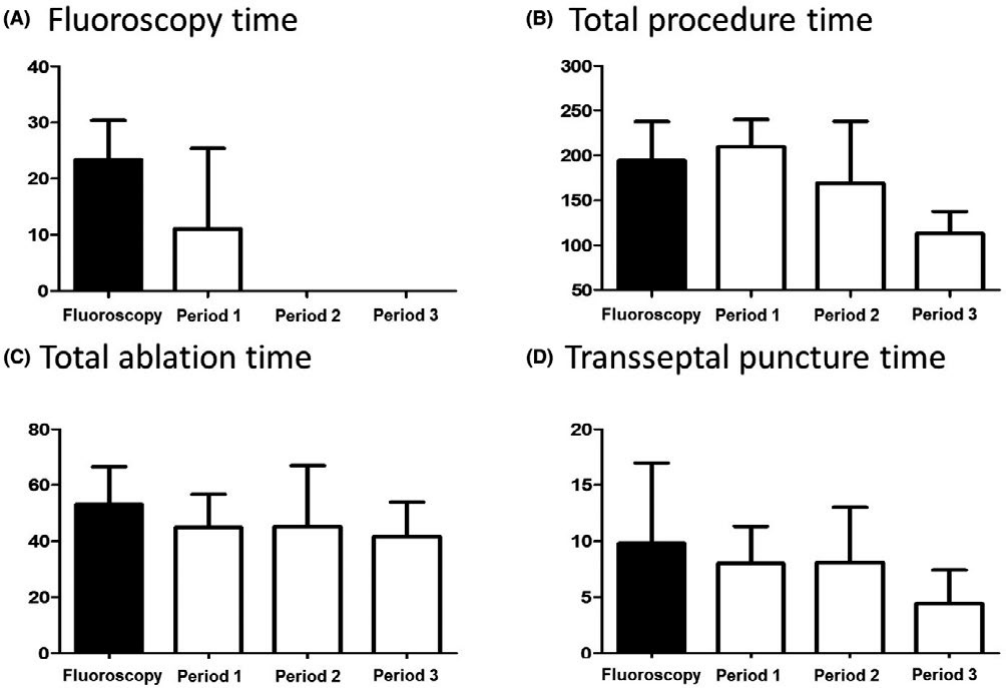
The comparisons of procedure time between fluoroscopy‐guided vs zero‐fluoroscopy‐guided period. Fluoroscopy time was complete zero during period 2 and 3. The total procedure time and transseptal puncture time were significantly decreased in period 3, although total ablation time was not different among groups

The total procedure time, total ablation time, transseptal puncture time, and fluoroscopy exposure time were significantly reduced after successfully changed from conventional fluoroscopy‐guided to zero‐fluoroscopy procedure (Figure 1).

**FIGURE 2:**
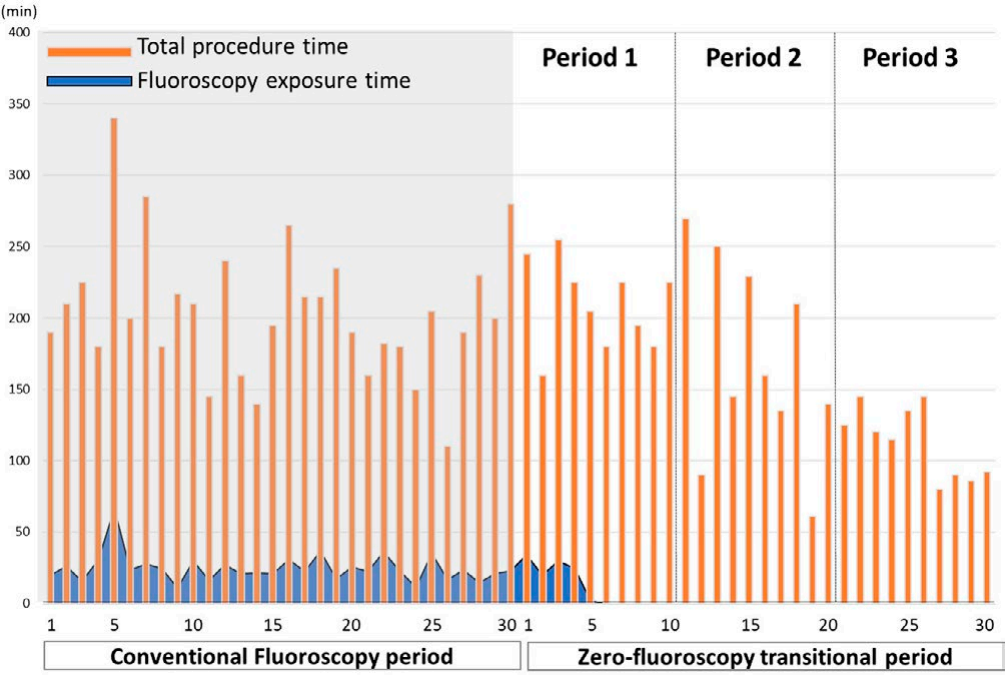
The serial total procedure and fluoroscopy time before and during zero‐fluoroscopy transition period. Although fluoroscopy equipment was always prepared to be able to use whenever necessary without restriction, fluoroscopy was used for only the first five patients in the zero‐fluoroscopy group. Total procedure time was continuously decreased, although there was time fluctuation in period 2

The completely zero‐fluoroscopy procedure was achieved at 6th case in zero‐fluoroscopy transitional period 1 (Figure 2). During period 2 and 3, there was no case using fluoroscopy (Figure 1A). The total procedure time has gradually decreased from period 1 to 3. It has a large fluctuation during period 2, but significantly decreased during period 3 compared to the fluoroscopy‐guided period (113.3 ± 24.8 minutes vs 194.4 ± 43.3 minutes, *P* < .001, Figure 1B). Total ablation time was slightly decreased during zero‐fluoroscopy transitional period, but there was no statistically significant difference among groups (Figure 1C). The average time for transseptal puncture was significantly decreased in period 3 compared to fluoroscopy‐guided period (Figure 1D).

## 4 DISCUSSION

In this study, we reported that a transition to completely zero‐fluoroscopy approach for AF catheter ablation could be performed by an experienced operator. Pulmonary vein isolation was achieved in all patients. The early transition period was relatively short, and there were only five cases that needed minimal fluoroscopy at the initial stage (period 1). The total procedure time during the study period rapidly decreased thereafter.

This approach has several advantages. First, the potential radiation hazard for both physicians and patients is completely none. Second, wearing a heavy lead apron during a procedure with a long duration in multiple cases can cause musculoskeletal disorders in the operators and physicians.^14^ Third, real‐time visualization of important structures (interatrial septum or coronary sinus) with intracardiac echocardiography seems to be beneficial for patient safety.

The main goal of an AF ablation procedure is pulmonary vein isolation, and it is sometimes necessary to perform adjunctive ablations.^11^ Fluoroscopy exposure may be useful for the various steps of the procedure, such as diagnostic catheter positioning, transseptal puncture, and complete pulmonary vein isolation. The recently developed techniques and modules have enhanced the feasibility of the fluoroless procedure and helped minimize the invasiveness including the radiation hazard. First, the 2D intracardiac ultrasound image integration with a 3D system (CARTO SOUND system) is useful not only for transseptal puncture but also for tagging important anatomical points such as the coronary sinus ostium, transseptal puncture site, and esophagus. Second, contact force‐ and ablation index‐guided ablation ensure optimal contact and prevent ablation lesion formation, as previously described in many studies.^15^, ^16^ Third, the ripple mapping technique enables the easy detection of residual potentials after pulmonary vein isolation. This mapping technique is also known to be helpful in defining activation through low‐voltage regions and to aid the ablation of atrial tachycardias.^17^

Recently, many studies have reported the feasibility and safety of the zero‐ or near‐zero fluoroscopy AF ablation procedure.^6^, ^10^, ^18^, ^19^ In a study by Sommer et al., a total of 1000 patients who underwent AF ablation between 2012 and 2017 were treated with a nonfluoroscopic approach and the overall complication rate was 2.0%, showing that the use of nonfluoroscopic catheter visualization technology is safe, and feasibled. In a complex case of a patient with cor triatriatum, the fluoroless technique was successfully applied for AF ablation.^20^ Novel approaches of performing the zero‐fluoroscopy procedure are frequently being introduced; for example, Guarguaqli et al. recently reported a novel technology for zero‐fluoroscopy AF ablation without ICE.^21^

Although the feasibility and safety profile of the zero‐fluoroscopy technique are continuously reported, there might be several situations when the use of fluoroscopy may be helpful or essential for the patient’s safety. First, correct positioning of the esophageal temperature probe needs fluoroscopic guidance. Although the ICE catheter shows the real‐time position of the esophagus and the temperature probe, the best way to place the catheter in the perfect position may be to use fluoroscopy. Second, the cardiac rotation or structure cannot be predicted before catheter advancement into the cardiac cavities in this zero‐fluoroscopy procedure. Therefore, we recommend preprocedural CT, which can identify the slope of the interatrial septum, abnormal structures inside the atrium‐like small pouches, or appendage thrombus before the procedure. Third, pericardial effusion can be detected easily from cardiac silhouette on fluoroscopy. We experienced one pericardial effusion case with zero‐fluoroscopy period with an unknown cause. We recommend to the use ICE for monitoring the pericardial complication during the procedure especially immediate after transseptal puncture or after radiofrequency ablation.

Most operators need time to be accustomed to new procedures and the effects of radiation do not appear immediately; thus, if the benefits are unclear, changing the procedure would be difficult because of the risks that may arise during the learning period. Fluoroscopy should also be used in situations in which it becomes necessary for the patient’s safety. Therefore, for unavoidable situations, we believe that applying the zero‐fluoroscopy technique in a setting in which fluoroscopy equipment is readily available can help reduce the radiation dosage without concerns about the patient’s safety. For the zero‐fluoroscopy procedure in this study, the learning period was considered to be relatively short, and completely zero‐fluoroscopy was achieved in only four cases after changing the protocol.

Radiation hazard should never be overlooked. With radiation exposure, the risk of cataract or dermatitis is elevated in interventional cardiologists and in patients.^3^, ^22^ The average patient dose for AF ablation is known to be 15 mV, and as a general rule of thumb, the absolute lifetime risk of fatal cancer for an adult increases by 0.05% for every 10 mSv of exposure.^1^, ^23^ The most active and experienced interventional cardiologists have a personal annual radiation dose exposure of about 5 mSv, which is three times higher than that of radiologists and nuclear physicians.^24^ Awareness of the risks associated with radiation exposure to patients and medical staff has significantly increased recently.^1^

This study has several limitations. First, this study was based on the experience of a single center that performs around 150 AF ablation procedures annually. Depending on previous experience, the difficulty of zero fluoroscopy may be different. According to our experience, practitioners who are experienced in ICE had little difficulty in transitioning to the zero‐fluoroscopy approach. Second, the number of cases is still small to confirm the safety of zero‐fluoroscopy procedure. The patient safety is the most important concern; thus, it is essential to prepare fluoroscopy equipment for its immediate use if the need arises. In the same sense, preprocedural imaging (CT or MRI scans) is useful. Also, in some cases, it is difficult to clearly identify the exact location of the wire tip in the vessel by utilizing only the sonography during the femoral vein puncture, and excessive manipulation could cause vascular complications. Therefore, the use of fluoroscopy should always be kept in mind during vascular access. Third, we cannot totally explain the cause of total procedure time reduction only with the decreased transseptal puncture time or fluoroscopy time. To find out the reason, the further study from another center or another operator should be performed.

### 4.1 Conclusion

During the transition period, complete zero‐fluoroscopy ablation of AF could be achieved safely and feasibly. The zero‐fluoroscopy technique decreased total procedure or septal puncture time significantly, saving the patients and physicians from radiation hazard. Fluoroscopy equipment backup and preprocedural imaging might be useful for the initial period of applying the zero‐fluoroscopy procedure.

## CONFLICT OF INTEREST

This study protocol was approved by the institutional review board that also waived the need for informed consent, and was conducted in accordance with the Declaration of Helsinki. (IRB no. 1901‐056‐1002/approval date: 16‐Jan‐2019).

## Supporting information

Fig S1‐S5

Video S1‐S2
