# Supplementary material for: Zero‐fluoroscopy catheter ablation for atrial fibrillation: a transitional period experience
Source: J Arrhythm. 2020 Oct 30;36(6):1061–7. doi: 10.1002/joa3.12448 (PMC7733568; doi:10.1002/joa3.12448)
Supplement: Supplementary file 1 — Fig S1‐S5 [file JOA3-36-1061-s001.pdf]

Supple Fig 1

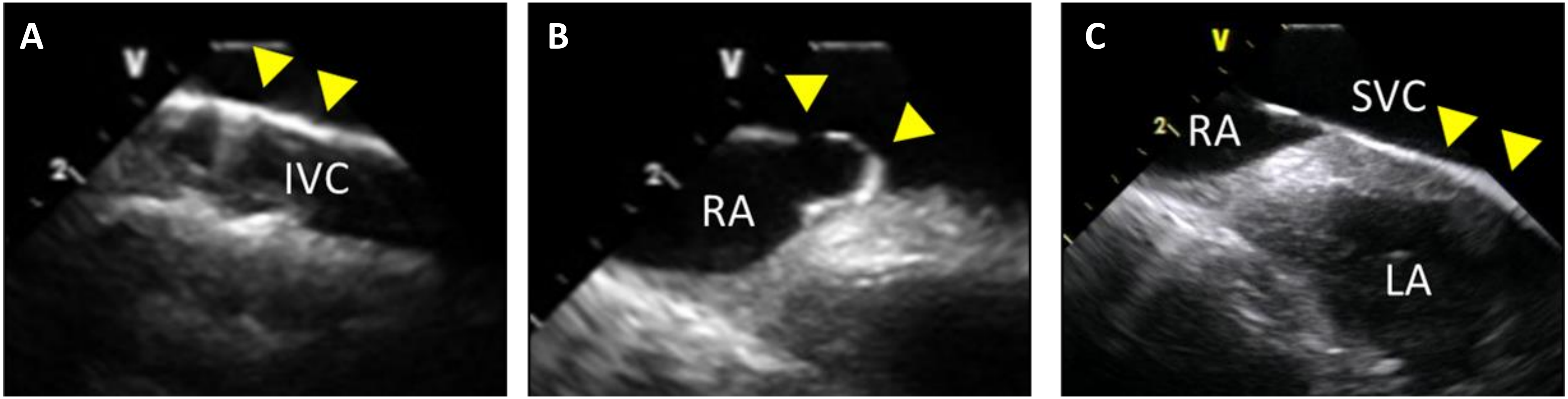

A long wire at (A) inferior vena cava (B) right atrium or (C) superior vena cava can be visualized with intracardiac echocardiography.

Supple Fig 2

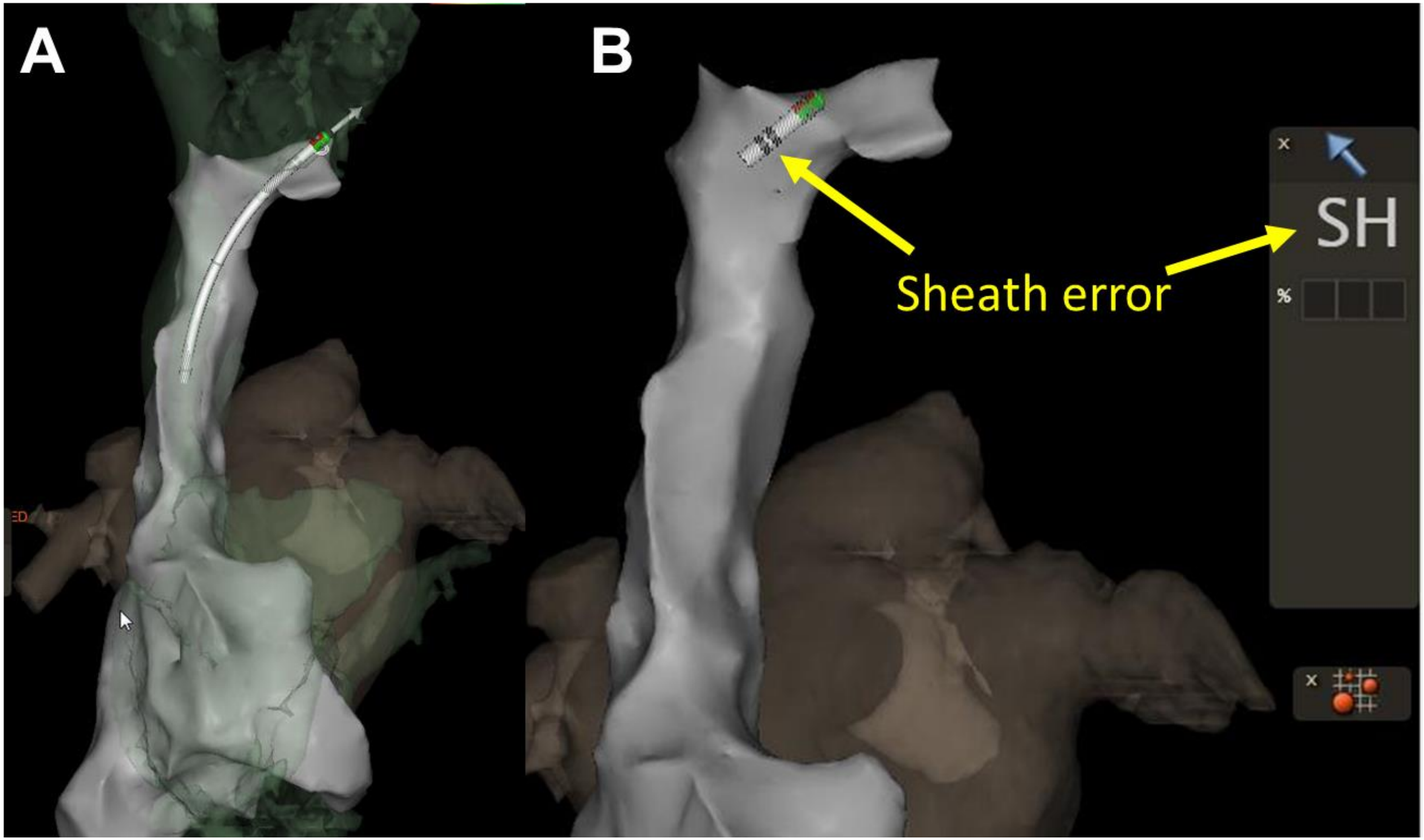

Supple Fig 3

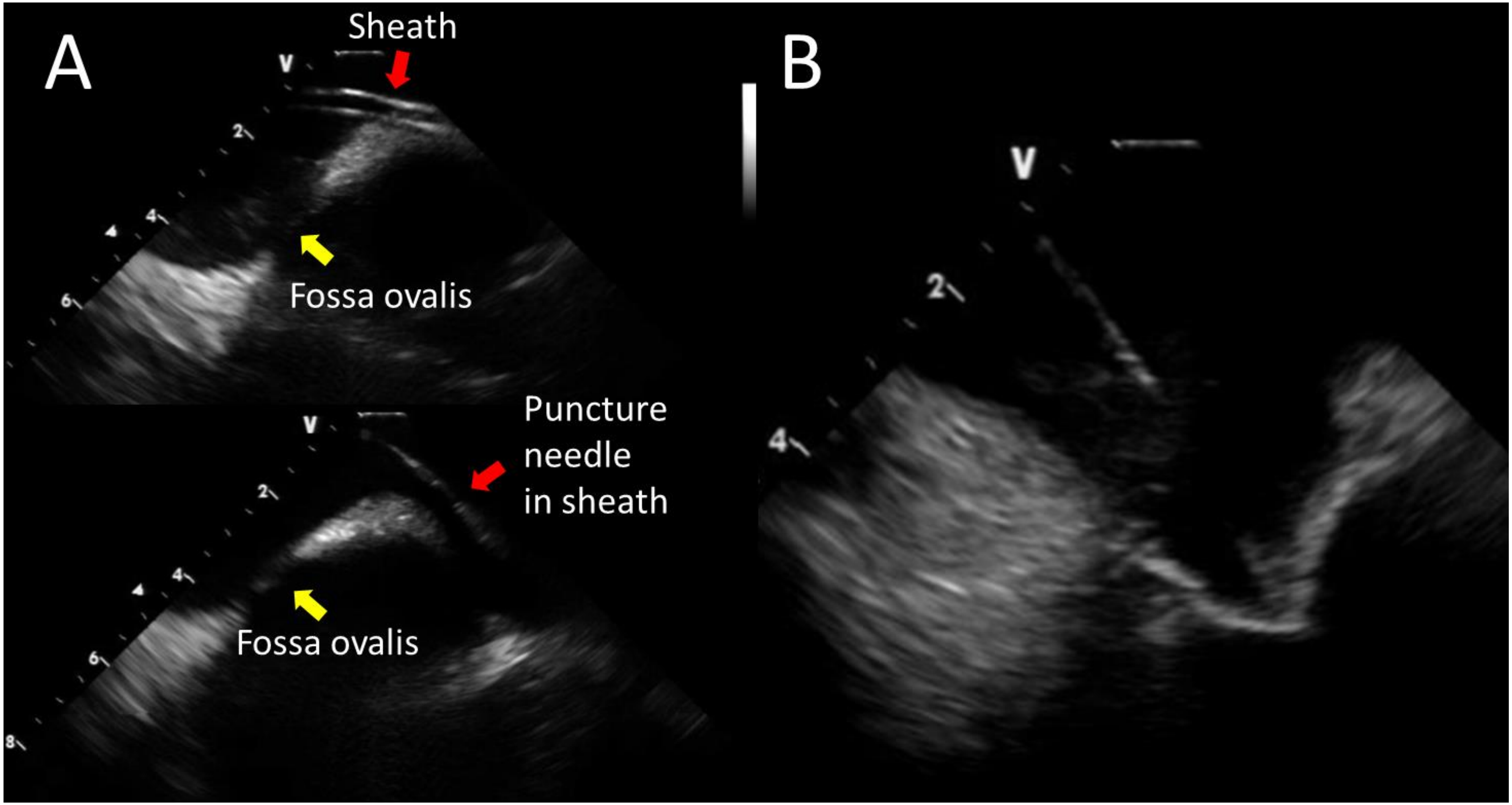
A
Sheath
Fossa ovalis
Puncture
needle
in sheath
Fossa ovalis
B

Supple Fig 4

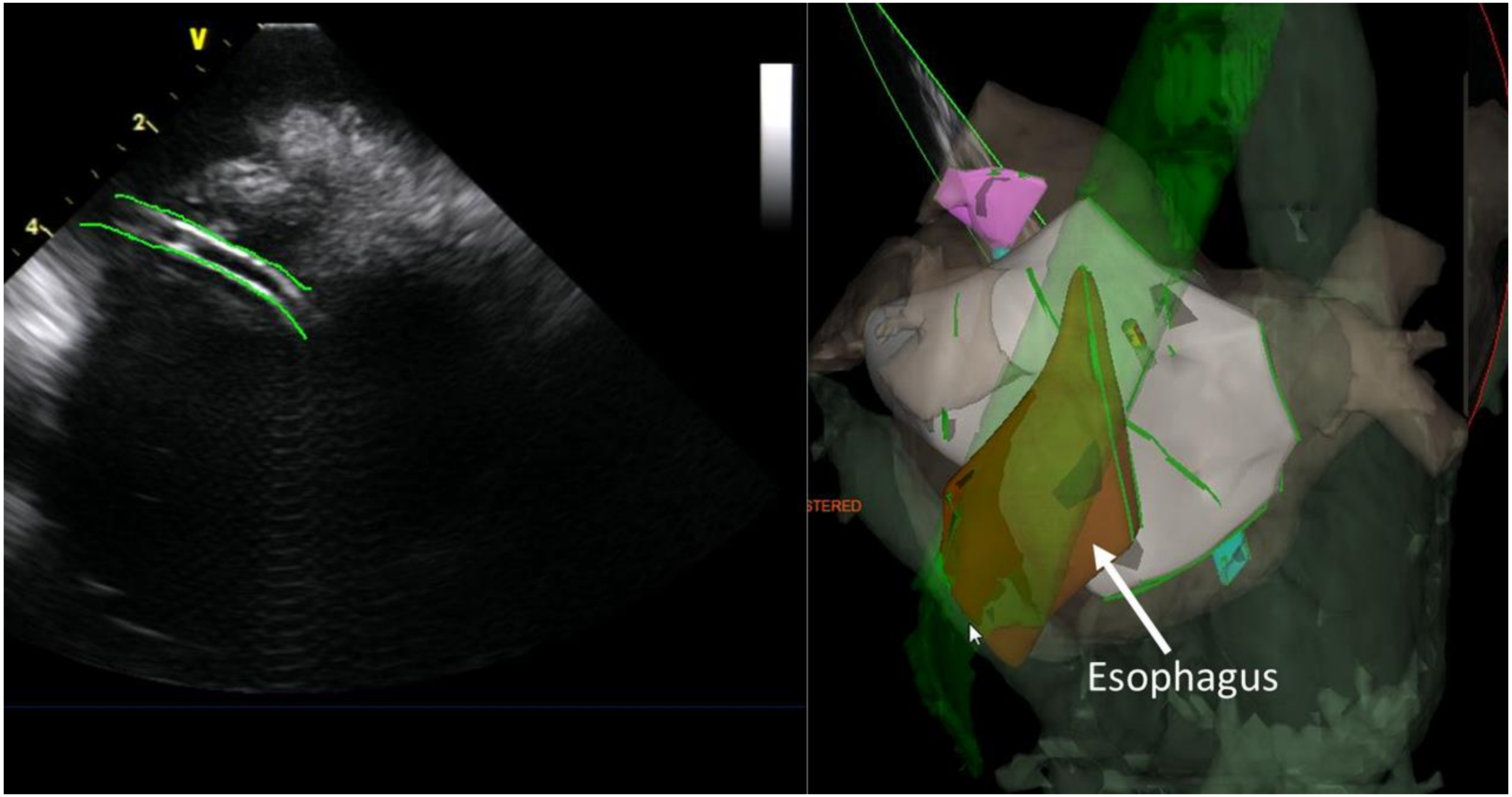

Supple Fig 5

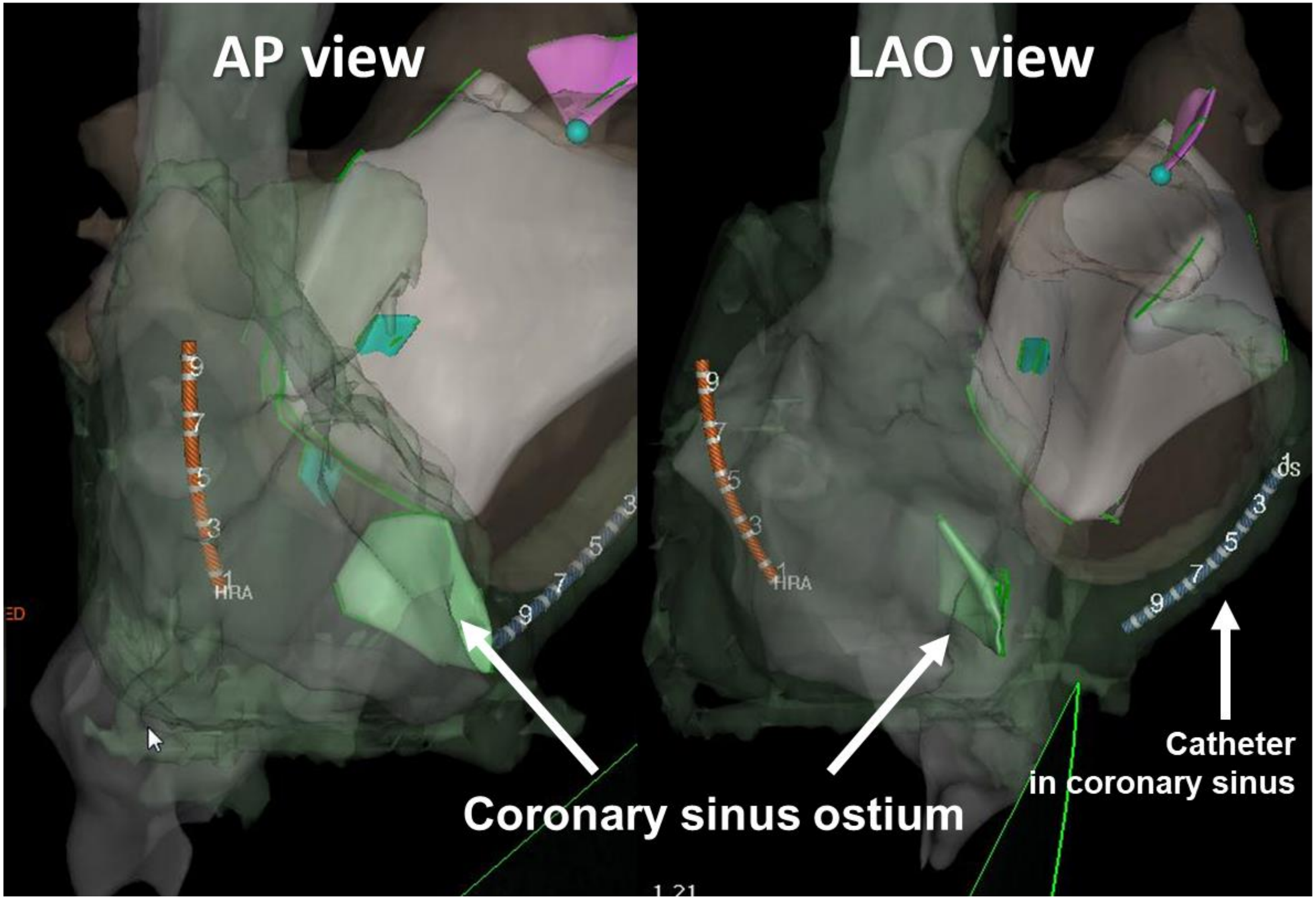
AP view
LAO view
9
7
5
3
1
HRA
ED
Coronary sinus ostium
Catheter
in coronary sinus
1.21
