# Supplementary figures and images for: Zero‐fluoroscopy catheter ablation for atrial fibrillation: a transitional period experience
Source: J Arrhythm. 2020 Oct 30;36(6):1061–7. doi: 10.1002/joa3.12448 (PMC7733568; doi:10.1002/joa3.12448)

## Slide 1
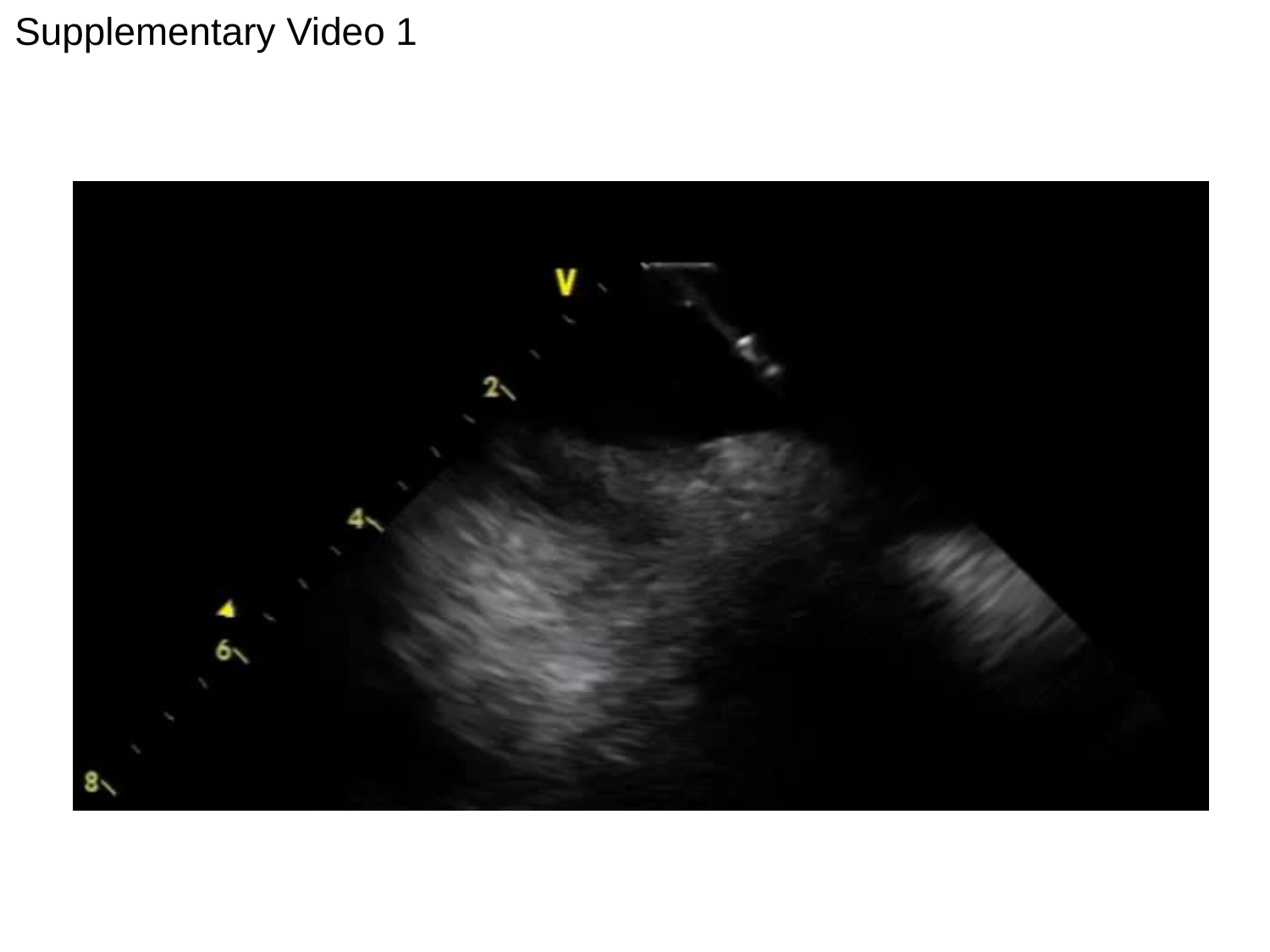

Supplementary Video 1

## Slide 2
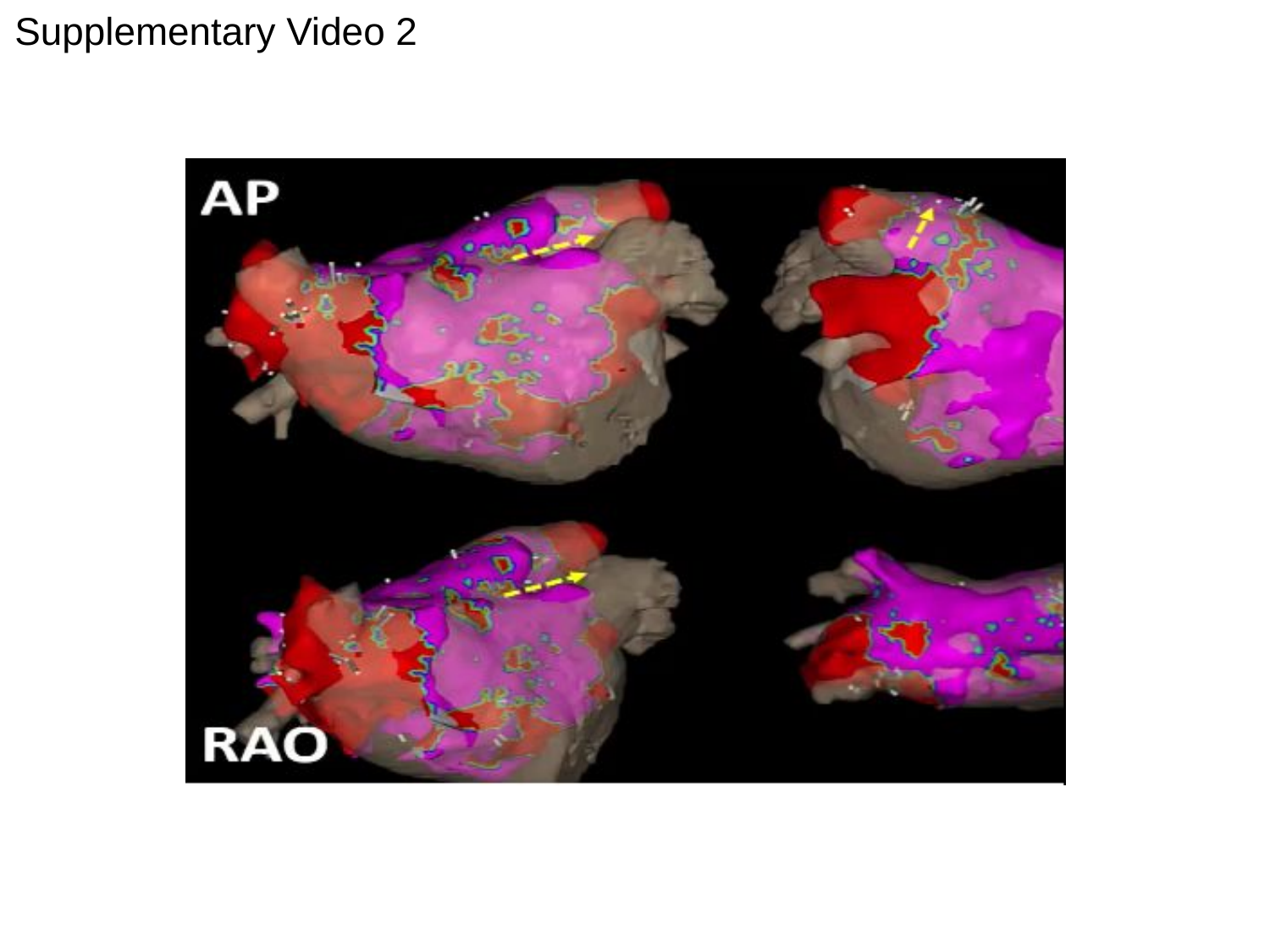

Supplementary Video 2

Supplement: Supplementary file 2 — Video S1‐S2 [file JOA3-36-1061-s002.pptx]
